# Measuring parent proxy-reported quality of life of 11 rare diseases in children in Zhejiang, China

**DOI:** 10.1186/s12955-020-01572-0

**Published:** 2020-11-23

**Authors:** Qisheng Gao, Shanshan Wang, Jianping Ren, Xin Wen

**Affiliations:** 1 Department of Public Health, Hangzhou Medical College, No. 481 Binwen Road, Hangzhou, 310053 Zhejiang Province China; 2 Department of Health Management, School of Medicine, Hangzhou Normal University, No. 2318 Yuhangtang Road, Hangzhou, 311121 Zhejiang Province China; 3 Center for Medical Science, Technology and Education of Zhejiang Province, No, 60 Hefang Street, Hangzhou, 310006 Zhejiang Province China

## Abstract

**Background:**

It has become increasingly important to measure the health-related quality of life (HRQoL) of rare diseases in children and adolescents in recent decades. Much attention has been paid to investigate the HROoL of a specific rare disease by self-report in previous studies. This study aimed to evaluate and compare the HROoL of 11 rare diseases in Chinese children by parent proxy-report, to explore the factors associated with HROoL of patients, and to understand the problems of most concern.

**Methods:**

A total of 651 children aged from 2 to 18 were enrolled from the Children’s Hospital Affiliated Zhejiang University in 2018. Their parents completed the parent proxy-report version of the Pediatric Quality of Life Inventory™ 4.0 (PedsQL™ 4.0). Independent samples *t*-test, one-way ANOVA, or Kruskal–Wallis *H* test was used to compare HROoL scores between groups. Multilevel linear regression models with random intercept were applied to analyze the relationship between socioeconomic variables and both the total score and subdomain scores.

**Results:**

The total PedsQL scores of Patent ductus arteriosus (PDA), Infantile agranulocytosis, Autoimmune thrombocytopenia (ITP), Polysyndactyly, Hirschsprung disease, Cleft lip and palate, Tetralogy of fallot, Myasthenia gravis, Guillain–barre syndrome, Glycogen storage disease, and Langerhans cell histiocytosis children were 79.65 ± 5.46, 95.88 ± 3.48, 71.39 ± 3.27, 91.77 ± 6.35, 76.18 ± 6.92, 96.33 ± 4.22, 77.85 ± 8.90, 95.99 ± 3.31, 85.77 ± 4.56, 82.97 ± 4.13 and 77.6 ± 5.15, respectively. Age was significantly associated with physical functioning, school functioning, and psychosocial health scores. The household registration place was significantly related to the total score. The most urgent desire of patients was to reduce the overall medical costs.

**Conclusions:**

This study showed that patients with PDA had the lowest physical functioning score, while patients with ITP scored the lowest in the emotional functioning, social functioning, school functioning, psychosocial health, and total scores. Incentive policies should be further adopted to improve orphan drug availability and reduce the economic burden of rare diseases.

## Background

Rare diseases, also known as “orphan diseases”, refer to diseases with a low prevalence but which are seriously debilitating or even life-threatening [1]. There is no universal definition of rare diseases worldwide. Different countries or regions have distinct definitions depending on disease incidence or prevalence, the severity of the disease, and the existence of adequate treatments or drugs. The average prevalence threshold used to define rare diseases across different organizations within individual jurisdictions ranges from 5 to 76 cases/100,000 people, with a global average prevalence threshold of 40 cases/100,000 people [2]. In China, rare diseases have not been officially defined until now. In 2010, experts in the seminar held by the Chinese Society of Genetic Medicine of the Chinese Medical Association reached a consensus that a rare disease was defined as the prevalence of less than 1/500,000 or neonatal morbidity of less than 1/10,000. It is estimated to exceed 16.8 million rare disease patients in China with a population of 1.4 billion according to this prevalence [3]. There are an estimated 6000–8000 rare diseases worldwide [4], 75% of rare diseases affect children, and 30% of rare diseases patients die before the age of 5 [5]. In 2018, the National Health Commission of China and other 4 government departments jointly formulated and published *China’s First List of Rare Diseases*, including 121 rare diseases [6]. The onset age of 43 rare diseases are during an infant and child’s stage, such as Albinism, Angelman syndrome, Arginase deficiency and so on. Most rare diseases are the result of small genetic changes and can severely impair physical, emotional, and mental abilities. These disabilities can decrease the quality of life considerably and cause a tremendous burden on the affected families and health care systems [7]. In recent decades, it has become increasingly important to measure the health-related quality of life (HRQoL) of rare diseases in children and adolescents. Generic instruments and disease-specific instruments may be applied to measure HRQoL in children and adolescents with the same rare diseases [8]. The generic instruments can measure HRQoL domains across diseases and can be used in different populations [9]. Among the generic instruments, the Pediatric Quality of Life Inventory™ (PedsQL™) is one of the widely used instruments in young people [10]. To evaluate patients’ HRQoL, many previous studies randomly selected healthy controls subjects for comparisons [11–14]. However, there is a paucity in the literature documenting differences in HRQoL among different rare diseases.

The main purpose of this study was to evaluate and compare HRQoL by surveying the parents of children with 11 different rare diseases between the ages of 2 and 18 by using the PedsQL™ instrument which can assess the domains outlined by WHO [15], to identify the association between potentially confounding factors on the HRQoL summary scores, and to understand the issues that patients are most concerned about.

## Methods

### Participants and procedures

The study was a cross-sectional, observational study performed in 2018 in the Children’s Hospital affiliated to Zhejiang University. The hospital we investigated is the highest-level children's hospital in the region, which can reduce misdiagnosis and missed diagnosis to some extent. Besides, the diagnosis of patients was confirmed by the doctor, and the screened cases were all hospitalized cases, so the accuracy of diagnosis was relatively high. We signed a confidentiality agreement with the hospital to ensure that the patient’s information will be kept strictly confidential. Patients diagnosed with rare diseases included in the *List of Rare Diseases in the European Union* during the period 2013–2017 were recruited from the hospital [16]. The criteria for inclusion were as follows: (1) The age of patients was between 2 and 18 years old; (2) The patients were diagnosed with no other diseases severely affecting the quality of life; (3) The parents were capable of understanding and expressing normally; (4) Consent of parents. The exclusion criteria were as follows: (1) Patients were younger than 2 years or older than 18 years at the time of interview; (2) Patients had other unrelated serious diseases. The parents were informed of the purpose, significance, and main content of the investigation orally before the telephone interview, and asked for their consent. The investigators were trained before the interview by the project manager to be very familiar with the questionnaire and use the normative expression. Besides, the investigators were responsible for ensuring that there were no missing data or logical errors in the questionnaire.

### Instruments

The Pediatric Quality of Life Inventory™ 4.0 (PedsQL™ 4.0), which includes parallel child self-report (age range 5–18 years) and parent proxy-report (age range 2–18 years), is a reliable, valid and sensitive instrument for widely used to assess HRQoL in the health and patient populations [17, 18]. While child self-report should be considered the gold standard for measuring HRQoL, in some cases, due to mental or cognitive impairment, or the lack of instruments for specific diseases, children may not be able to complete the questionnaire directly, and parent proxy-report may be a strategy for assessing children or adolescents’ HRQoL [19, 20]. The items for child self-report and parent proxy-report are basically consistent, except that they differ in the developmentally appropriate language and the first or third person tense [17, 21]. Previous empirical studies have confirmed the validity of parental reports [22, 23]. Besides, it is generally the parents’ perceptions of their children’s HRQoL that influence healthcare utilization [24, 25]. In this study, the Chinese parent proxy-report version of the PedsQL™ 4.0 which has been validated in Chinese children was applied to evaluate children’s quality of life [26–29]. This scale consists of 23 items, which are divided into 4 dimensions: Physical functioning (8 items), Emotional functioning (5 items), Social functioning (5 items) and School functioning (5 items). The latter 3 dimensions can also be united, called psychosocial health. Each item was scored using a 5-point response scale, of which 0 = never a problem, 1 = almost never a problem, 2 = sometime a problem, 3 = often a problem, 4 = almost always a problem. The items were reverse-scored and linearly converted to a score of 0–100 (0 = 100, 1 = 75, 2 = 50, 3 = 25, 4 = 0) with higher scores indicated better HRQoL. The scale scores were computed as a sum of the items divided by the number of items answered. If more than 50% of the items in the scale were missing, the scale score would not be calculated. The physical, emotional, social, school, psychosocial, and total scores were used in this study. In addition to PedsQL™ 4.0, all parents were required to complete a brief questionnaire covering the children’s socio-demographic characteristics, such as gender, age group, household registration place, household type, and monthly family income per capita.

**Fig. 1:**
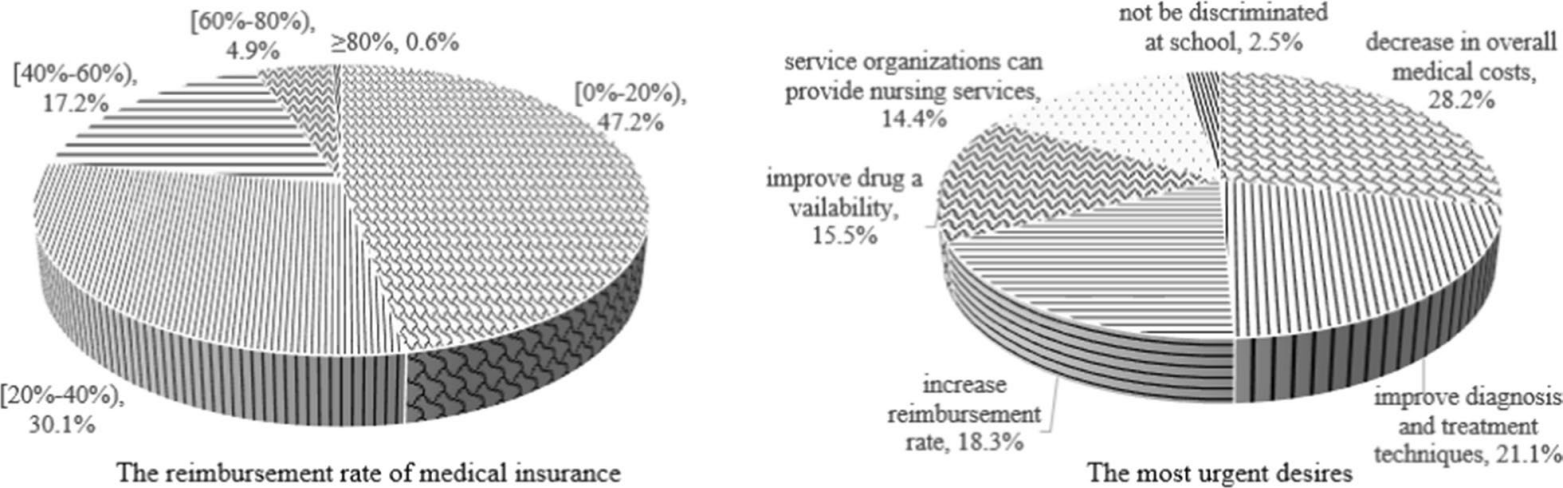
The choice proportions of the reimbursement rate of medical insurance and the most urgent desires

### Statistical analysis

Categorical data were presented as counts and percentages. Quantitative data were described by mean and standard deviation. The distribution of the HRQoL scores was tested for normality using the Shapiro–Wilk test. Comparisons between two groups were made using independent *t-*test. When there were more than two groups, the differences were evaluated by one-way ANOVA and Kruskal–Wallis *H* test according to the homogeneity of variance.

Multiple comparisons were made by Dunn’s post hoc test or least significant difference test (LSD). The multilevel linear regression models with random intercept were applied to analyze the relationship between socioeconomic variables and total score and subdomain scores. Initially, a model was estimated with the intercept only to estimate the proportion of variance due to the diseases concerning the individuals. This model served as the basis for evaluating the reduction in the variance of other models studied. After this, individual socioeconomic variables were tested. The two-tailed *P* < 0.05 was considered to be statistically significant. Data were processed and analyzed using R 3.6.1 for windows.

## Results

### Participants’ characteristics

A total of 651 children’s parents completed the questionnaire. The socio-demographic characteristics of their children were presented in Table 1. The most frequently presenting rare disease was ITP (18.4%). Among the children, 373 (57.3%) were male, 310 (47.6%) aged between 2 to 4 years, 474 (72.8%) came from urban areas, and 288 (44.2%) of the monthly family income per capita less than 5000 CNY. These children came from 9 cities in Zhejiang province. They were 350 (53.8%) from Hangzhou, 105 (16.1%) from Ningbo, 67 (10.3%) from Shaoxing, 25 (3.8%) from Wenzhou, 51 (7.8%) from Jinhua, 10 (1.5%) from Lishui, 29 (4.5%) from Quzhou, 11 (1.7%) from Taizhou and 3 (0.5%) from Zhoushan. Due to the discrepancy of sample size in different cities, except the provincial capital city of Hangzhou, the other 8 cities were integrated into a group.

**Table 1:** Socio-demographic characteristics of the total participants

Variables	Frequency (%)
Types of rare diseases	
Patent ductus arteriosus	40 (6.1)
Infantile agranulocytosis	67 (10.3)
Autoimmune thrombocytopenia	120 (18.4)
Polysyndactyly	66 (10.1)
Hirschsprung disease	118 (18.1)
Cleft lip and palate	51 (7.8)
Tetralogy of fallot	56 (8.6)
Myasthenia gravis	42 (6.5)
Guillain–barre syndrome	33 (5.1)
Glycogen storage disease	30 (4.6)
Langerhans cell histiocytosis	28 (4.3)
Gender	
Male	373 (57.3)
Female	278 (42.7)
Age group	
2–4 year	310 (47.6)
5–7 year	220 (33.8)
8–12 year	103 (15.8)
13–18 year	18 (2.8)
Household registration place	
Hangzhou	350 (53.8)
Other cities	301 (46.2)
Household type	
Urban areas	474 (72.8)
Rural areas	177 (27.2)
Family monthly income per capita	
< ¥5000	288 (44.2)
¥5000–¥10,000	259 (39.8)
> ¥10,000	104 (16.0)
Total	651 (100)

### PedsQL scores of children

Univariate analysis of categorical variables relative to PedsQL total score and subdomain scores were summarized in Table 2. There were significant differences in the total scale and each subscale in different rare diseases, household registration place, and monthly family income per capita (all *P* < 0.05).The physical functioning score of children with Patent ductus arteriosus (PDA) was significantly lower than that of children with Infantile agranulocytosis, Polysyndactyly, Hirschsprung disease, Cleft lip and palate, and Myasthenia gravis (all *P* < 0.05). The emotional functioning, school functioning and total scores of ITP were significantly lower than those of other rare diseases except Langerhans cell histiocytosis (all *P* < 0.05). The social functioning score of ITP was significantly lower than that of other rare diseases except Hirschsprung disease (all *P* < 0.001), and the psychosocial health score of ITP was significantly lower than that of other rare diseases (all *P* < 0.001). Females reported significantly higher scores than male in the physical functioning score (*t* = − 3.29, *P* = 0.001) and total score (*t* = − 2.43, *P* = 0.015). The 2–4 year group was significantly higher than the 8–12 year group in the emotional functioning score (*t* = 2.917, *P* = 0.004). The 2–4 year group was significantly higher than the 5–7 year group (*Z* = 2.979, *P* = 0.017) and the 8–12 year group (*Z* = 5.512, *P* < 0.001) in the school functioning score. The 8–12 year group was significantly lower than other age groups in the psychosocial health score (all *P* < 0.05). The 2–4 year group was significantly higher than the 8–12 year group (*Z* = 3.436, *P* = 0.004) in the total score. The scores of subscale and total scale of children with household registration place in Hangzhou were higher than those of children with household registration place in other cities (all *P* < 0.001). Children in urban areas reported significantly higher scores than children in rural areas in physical functioning score (*t* = 3.31, *P* = 0.001), school functioning score (*t* = 3.24, *P* = 0.001),psychosocial health score (*t* = 2.45, *P* = 0.015) and total score (*t* = 3.05, *P* = 0.002). The total score and each subdomain score of the children with family monthly income per capita over 10,000 CNY were significantly higher than those of the low-income groups (all *P* < 0.05).

**Table 2:** Comparisons of PedsQL scores reported by parents

Variables	Physical functioning	Emotional functioning	Social functioning	School functioning	Psychosocial health	Total
Types of rare diseases						
Patent ductus arteriosus	65.86 ± 8.88	84.38 ± 8.18	87.75 ± 8.91	88.88 ± 8.95	87.00 ± 6.16	79.65 ± 5.46
Infantile agranulocytosis	97.01 ± 3.86	94.93 ± 6.18	97.76 ± 5.02	93.13 ± 9.8	95.27 ± 4.55	95.88 ± 3.48
Autoimmune thrombocytopenia	73.33 ± 4.72	73.04 ± 6.16	75.75 ± 3.47	62.25 ± 7.21	70.35 ± 3.81	71.39 ± 3.27
Polysyndactyly	90.39 ± 9.76	91.59 ± 9.49	94.09 ± 7.99	91.82 ± 9.27	92.50 ± 6.78	91.77 ± 6.35
Hirschsprung disease	74.84 ± 10.44	78.56 ± 8.80	77.84 ± 10.16	74.28 ± 10.38	76.89 ± 7.20	76.18 ± 6.92
Cleft lip and palate	99.02 ± 2.38	96.47 ± 6.35	92.75 ± 11.42	95.49 ± 7.09	94.9 ± 6.160	96.33 ± 4.22
Tetralogy of fallot	72.38 ± 11.8	80.54 ± 10.77	84.38 ± 11.64	77.41 ± 9.72	80.77 ± 8.72	77.85 ± 8.90
Myasthenia gravis	96.58 ± 4.72	94.29 ± 5.90	98.69 ± 3.83	94.05 ± 7.90	95.67 ± 3.97	95.99 ± 3.31
Guillain–barre syndrome	74.34 ± 10.39	86.21 ± 8.48	96.52 ± 5.23	92.88 ± 4.68	91.87 ± 3.25	85.77 ± 4.56
Glycogen storage disease	76.15 ± 6.69	85.50 ± 9.04	90.33 ± 6.81	84.00 ± 8.14	86.61 ± 4.58	82.97 ± 4.13
Langerhans cell histiocytosis	73.10 ± 10.74	79.29 ± 11.44	90.89 ± 8.50	69.82 ± 8.44	80.00 ± 5.72	77.60 ± 5.15
*χ*^2^	400.95*	338.69*	371.83*	430.23*	471.05*	483.00*
*P*	< 0.001	< 0.001	< 0.001	< 0.001	< 0.001	< 0.001
Sex						
Male	79.41 ± 13.45	83.67 ± 11.24	86.42 ± 11.63	80.44 ± 14.72	83.51 ± 10.86	82.08 ± 10.51
Female	82.94 ± 13.65	84.98 ± 11.85	87.59 ± 11.77	81.94 ± 15	84.84 ± 11.25	84.18 ± 11.12
*t*	− 3.29	− 1.44	− 1.26	− 1.28	− 1.52	− 2.43
*P*	0.001	0.151	0.207	0.202	0.130	0.015
Age group						
2–4 year	81.99 ± 14.39	85.45 ± 11.5	86.87 ± 12.43	83.92 ± 13.81	85.41 ± 10.78	84.22 ± 10.98
5–7 year	80.24 ± 13.08	83.64 ± 11.87	87.11 ± 10.86	79.93 ± 15.59	83.56 ± 11.35	82.41 ± 10.85
8–12 year	79.58 ± 12.63	81.65 ± 10.72	85.53 ± 11.18	74.76 ± 14.41	80.65 ± 10.73	80.28 ± 10.19
13–18 year	78.30 ± 11.96	85.28 ± 9.31	93.33 ± 9.70	82.50 ± 13.2	87.04 ± 9.07	84.00 ± 8.03
*F* (*χ*^2^)	3.78*	3.16	2.32	32.11*	5.53	13.36*
*P*	0.287	0.024	0.074	< 0.001	0.001	0.004
Household registration place						
Hangzhou	85.24 ± 13.48	86.43 ± 11.96	89.49 ± 11.04	84.14 ± 14.58	86.69 ± 11.08	86.18 ± 11.13
Other cities	75.88 ± 12.01	81.68 ± 10.42	83.94 ± 11.74	77.52 ± 14.38	81.05 ± 10.2	79.25 ± 9.14
*t*	9.37	5.42	6.21	5.81	6.72	8.73
*P*	< 0.001	< 0.001	< 0.001	< 0.001	< 0.001	< 0.001
Household types						
Urban areas	81.96 ± 13.72	84.66 ± 11.59	87.28 ± 11.82	82.23 ± 14.56	84.72 ± 10.95	83.76 ± 10.89
Rural areas	78.11 ± 13.06	83.08 ± 11.27	85.96 ± 11.33	78.02 ± 15.22	82.35 ± 11.12	80.88 ± 10.35
*t*	3.31	1.56	1.28	3.24	2.45	3.05
*P*	0.001	0.119	0.201	0.001	0.015	0.002
Family monthly income per capita						
< ¥5000	79.42 ± 13.79	83.44 ± 10.95	86.46 ± 11.69	81.77 ± 13.55	83.89 ± 10.32	82.33 ± 10.26
< ¥5000–¥10,000	79.72 ± 12.82	83.53 ± 11.78	85.6 ± 11.69	78.71 ± 15.69	82.61 ± 11.48	81.61 ± 10.74
> ¥10,000	88.04 ± 13.08	88.17 ± 11.68	91.49 ± 10.68	85.1 ± 15.18	88.25 ± 10.91	88.18 ± 11.08
*F* (*χ*^2^)	17.81	14.352*	20.343	15.451*	20.405*	15.26
*P*	< 0.001	0.001	< 0.001	< 0.001	< 0.001	< 0.001

^*^χ^2^ of Kruskal–Wallis *H* test

### Factors associated with HRQoL

The multilevel linear regression models were shown in Table 3. The physical functioning score of 8–12-year-old children was higher than that of 2–4-year-old children (*P* < 0.001). The score of school functioning decreased with the increase of children’s age (*P* < 0.05). The psychosocial health score of 8–12-year-old children was lower than that of 2–4-year-old children (*P* = 0.019). The total score of children whose household registration place was in Hangzhou was higher than that of children in other cities (*P* = 0.027).

**Table 3:** Multilevel regression coefficients and *P* value between variables and PedsQL scores

	Physical functioning		Emotional functioning		Social functioning		School functioning		Psychosocial health		Total	
	*β* (SE)	*p* value	*β* (SE)	*p* value	*β* (SE)	*p* value	*β* (SE)	*p* value	*β* (SE)	*p* value	*β* (SE)	*p* value
Intercept	80.85 (3.82)	< 0.001	85.62 (2.45)	< 0.001	90.99 (2.37)	< 0.001	86.79 (3.46)	< 0.001	87.92 (2.59)	< 0.001	85.58 (2.74)	< 0.001
Sex (ref = Male)												
Female	0.25 (0.51)	0.619	− 0.75 (0.65)	0.247	− 0.23 (0.59)	0.692	− 0.64 (0.64)	0.317	− 0.83 (0.43)	0.057	− 0.74 (0.38)	0.051
Age group (ref = 2–4 year)												
5–7 year	1.10 (0.64)	0.084	0.07 (0.82)	0.936	− 0.47 (0.74)	0.530	− 2.46 (0.81)	0.002	− 1.01 (0.55)	0.065	− 0.05 (0.48)	0.921
8–12 year	3.04 (0.85)	< 0.001	0.26 (1.06)	0.804	− 0.74 (0.97)	0.445	− 4.50 (1.05)	< 0.001	− 1.67 (0.71)	0.019	0.15 (0.62)	0.808
13–18 year	1.09 (1.66)	0.514	− 0.29 (2.09)	0.889	0.26 (1.90)	0.891	− 6.66 (2.07)	0.001	− 2.15 (1.40)	0.125	− 1.45 (1.22)	0.236
Household registration place (ref = Hangzhou)												
Other cities	− 0.79 (0.56)	0.158	− 0.11 (0.71)	0.882	− 0.94 (0.65)	0.152	− 1.28 (0.71)	0.070	− 0.57 (0.48)	0.235	− 0.93 (0.42)	0.027
Household type (ref = cities and towns)												
Rural areas	− 0.99 (0.59)	0.096	0.82 (0.75)	0.279	0.69 (0.69)	0.320	− 0.44 (0.75)	0.555	− 0.07 (0.51)	0.895	− 0.58 (0.44)	0.191
Family monthly income per capita (ref = < ¥5000)												
¥5000–¥10,000	− 1.05 (0.61)	0.084	0.76 (0.78)	0.328	− 1.11 (0.71)	0.117	0.25 (0.78)	0.748	0.06 (0.53)	0.909	− 0.14 (0.46)	0.761
> ¥10,000	0.50 (0.81)	0.538	1.12 (1.02)	0.276	1.00 (0.94)	0.287	1.88 (1.02)	0.064	1.20 (0.69)	0.081	0.95 (0.60)	0.113
Conditional *R*^2^	0.812		0.486		0.523		0.678		0.719		0.792	

### Reimbursement rates and the most urgent desires

The reimbursement rates of medical insurance for rare diseases were set to four grades: 0–20%, 20–40%, 40–60%, 60–80%, ≥ 80%. The proportion of patient who chose these four levels were 47.2%, 30.1%, 17.2%, 4.9% and 0.6%, respectively. The choice proportions of the most urgent desires were 28.2%,21.1%,18.3%,15.5%,14.4%, and 2.5% for a decrease in overall medical costs, improve diagnosis and treatment techniques, increase reimbursement rate, improve drug availability, service organizations can provide nursing services, not be discriminated at school, respectively (Fig. 1).

## Discussion

In the current study, we used the parent proxy-report version of the PedsQL™ 4.0 instrument to assess and compare the quality of life of patients with 11 rare diseases. Many studies proved that there was no statistically significant difference between child self-report and parent-proxy report of the PedsQL™ 4.0 scales, and moderate to good concordance was found between the child and parent-proxy scores [8, 15, 30–33]. However, other studies showed inconsistencies between parent-proxy and child self-report of HRQoL, especially in the subjective domains such as emotional and social functioning [23, 34–36]. Collins et al*.* pointed out that parent-proxy questionnaires may potentially reduce the accuracy of children’s experience [37]. Most authors agree that parent-proxy report should be used as a supplement to child self-report as a secondary outcome measure [15].

The present study demonstrated that PDA had the lowest score in the physical functioning, ITP scored the lowest in the emotional functioning, social functioning, school functioning, psychosocial health and total score. ITP is an acquired autoimmune disease associated with some symptoms, such as spontaneous bruising, mucosal bleeding, epistaxis, and even fatal bleeding events [32]. The low quality of life of ITP may be associated with incessant worries of unpredictable bleeding, fears of invasive procedures, and risk of splenectomy. Additionally, the serious side effects of corticosteroid therapy and daily life restrictions may worsen it [38, 39]. Sood et al. summarized that the average scores of physical functioning, psychosocial health and total score measured by parent proxy-report version of PedsQL™ 4.0 for 11–18 year patients with Hirschsprung disease were 89.17 (SD = 14.40), 82.53 (SD = 17.61) and 84.84 (SD = 14.91), respectively [8], which were higher the results revealed by the current study and Collins [37] who reported that the psychosocial health score of children with Hirschsprung disease aged between 2 and 10 years (mean = 76.0) was significantly less than the healthy control group (mean = 81.2). Kwon et al. reported that the average scores of physical functioning, emotional functioning, social functioning, school functioning and total score measured by parent proxy-report version of PedsQL™ 4.0 of patients with Repaired tetralogy of fallot from 8.4 to 18.7 years old were 78.00 (SD = 19.30),76.00 (SD = 17.60),73.00 (SD = 22.40), 73.50 (SD = 17.90) and 77.40 (SD = 15.00), respectively [40], which were significantly lower than the normal group except the emotional functioning score, and the total score was very close to the present study. Collett et al*.* found that the scores of physical functioning and psychosocial health of children with Orofacial Clefts were 85.68 (SD = 17.57) and 81.03 (SD = 15.20), which were lower than the results of this study, and negligible differences were found in psychosocial outcomes between children with and without orofacial clefts [41]. Storch et al*.* reported that the average scores of physical functioning, emotional functioning, social functioning, school functioning, psychosocial health and total score measured by parent proxy-report version of PedsQL™ 4.0 of patients aged from 3 to 25 years with Glycogen storage disease Type I were 76.45 (SD = 19.63), 76.93 (SD = 19.24),74.23 (SD = 20.33), 71.92 (SD = 18.28), 74.36 (SD = 15.07) and 74.88 (SD = 15.64), respectively, which were all lower than that of the current study except for the physical functioning score [14], and the healthy control sample was significantly higher than the GSD sample in these domains.

HRQoL is a broad multidimensional concept influenced by numerous factors. The results of multilevel linear regression showed that children at the age of 8–12 reported higher physical functioning scores and less psychosocial health scores than children who at the age of 2–4. Although there was no significant difference among the 13–18 age group, 5–7 age group and 2–4 age group, the regression coefficients indicated that the score of psychosocial health decreased with age. Collins et al. reported a similar result that psychosocial functioning in the children with Hirschsprung disease was negatively affected by increasing age [37]. Peer teasing has been shown to be a strong predictor of self-concept and parent-reported behavior problems [42]. Collett et al.found that many pre-school children with Orofacial clefts are quite resilient, despite the potential for social stigmatization. However, as children develop and peer groups become more important, this situation may become more difficult to maintain [41]. This study also showed that increasing age negatively affected school functioning scores. Miatton et al.revealed that children with Tetralogy of fallot showed significantly lower scores on the school performances than healthy peers [43]. Roodbol et al. reported that many children were held back in class or dropped out of school after developing Guillain-Barré syndrome [44]. Khalil found that age at the time of surgery was negatively affecting school functioning (β = − 0.907) in children with Hirschsprung’s disease [45]. The results of the multivariate analysis show that gender was not an influencing factor of HRQol. Though Mills et al. found that girls with Hirschsprung’s disease had higher scores across all HRQol scales, the difference was not significant [33]. Many previous studies also indicated that gender did not have a significant effect on aspects of the global HRQol in children with Hirschsprung's disease [8, 37, 45]. The total score of children whose household registration place was in Hangzhou was significantly higher than that in other cities, which is likely to be the provincial capital city with the highest per capita GDP, representing a higher socio-economic status, where children have access to better medical services and education, and develop positive coping mechanisms to cope with adversity. Moreover, Damiano et al. reported that in families with higher income, patients with Cleft lip and palate had better physical, psychosocial, and total health scores [46].

A 2016 study investigated 1771 patients covered with 142 rare diseases in China. According to the study, 66% of the patients had been misdiagnosed before, and the most serious problem in the course of treatment was high medical cost (32.07%).Other problems were few types of drugs and rehabilitation (15.81%), poor treatment effect (12.25%), low reimbursement rate (12.14%), poor accessibility to health service (11.80%), respectively [47]. Among the 121 rare diseases listed in *China’s First List of Rare Diseases*, 9 rare diseases have no medication, and the related medications of 13 rare diseases are excluded from the coverage of medical insurance [48]. Another study revealed similar results to the present study that the reimbursement rate of medical insurance was between 10 to 50%, and 80% of patients did not have commercial insurance [49]. Therefore, the Chinese government should strengthen its support to carry out fundamental research on the treatment and drugs of rare diseases, and formulate and implement incentive policies for the production or importation of orphan drugs. Moreover, it is recommended to include more rare disease drugs in medical insurance and raise the reimbursement rate to enhance orphan drug availability and reduce the financial burden of rare diseases.

## Limitations

Some limitations of the study should be considered. First, we did not inquire about the age at onset of symptoms or age at diagnosis. Although most of the literature did not take the age at onset of symptoms or age at diagnosis as an influencing factor, we still believe that this is one of the limitations of our study. Second, the research team did not confirm the diagnosis through a manual chart review, and there may be misdiagnosis. Third, since there were only 18 subjects in the 13–18 age group, this may be underrepresented in our sample. Fourth, according to the information we collected, among the 11 rare diseases included in the study, only PDA, Polysyndactyly, Cleft lip and palate can be cured by surgery, and the rest are treated with symptomatic support or no treatment. There is no denying that different treatments and prognosis will affect the quality of life. If patients with rare diseases can be cured by surgery, their quality of life will theoretically be improved in the long run. For most rare diseases that lack specific treatment, the improvement of quality of life by general supportive treatment requires further scientific evaluation. However, since we did not investigate information about treatment and prognosis, we are unable to provide evidence of the effect of treatment on the quality of life of rare diseases, which will be the direction of our future efforts.

## Conclusions

This study revealed that the HRQoL of children with ITP was relatively lower compared with other rare diseases. We should pay more attention to the children’s mental health and school functioning with age. Patients still face a lot of problems when seeking treatment, such as a lack of effective medicine and unable to afford medical expenses. Therefore, further measures should be adopted to improve the efficiency of diagnosis and the effect of treatment, to improve the availability of health services, and to alleviate the economic burden.

## Data Availability

Please contact author for data requests.

## Abbreviations

HRQoL: Health-related quality of life
PedsQL™: Pediatric quality of life inventory™
LSD: Least significant difference
ITP: Autoimmune thrombocytopenia
PDA: Patent ductus arteriosus
GSD: Glycogen storage disease
